# Confounding in association studies: month of birth and multiple sclerosis

**DOI:** 10.1007/s00415-014-7241-y

**Published:** 2014-01-12

**Authors:** Barnaby Fiddes, James Wason, Stephen Sawcer

**Affiliations:** 1 Department of Clinical Neurosciences, University of Cambridge, Cambridge Biomedical Campus, Hills Road, Cambridge, CB2 0QQ UK; 2 Medical Research Council Biostatistics Unit, Cambridge, CB2 0SR UK

**Keywords:** Confounding, Association, Multiple sclerosis, Month of birth

## Abstract

Association studies form the backbone of biomedical research, with almost every effort in the field ultimately boiling down to a comparison between groups, coupled with some form of statistical test intended to determine whether or not any observed difference is more or less than would be expected by chance. Unfortunately, although the paradigm is powerful and frequently effective, it is often forgotten that false positive association can easily arise if there is any bias or systematic difference in the way in which study subjects are selected into the considered groups. To protect against such confounding, researchers generally try to match cases and controls for extraneous variables thought to correlate with the exposures of interest. However, if seemingly homogenously distributed exposures are actually more heterogeneous than appreciated, then matching may be inadequate and false positive results can still arise. In this review, we will illustrate these fundamental issues by considering the previously proposed relationship between month of birth and multiple sclerosis. This much discussed but false positive association serves as a reminder of just how heterogeneous even easily measured environmental risk factors can be, and how easily case control studies can be confounded by seemingly minor differences in ascertainment.

**Electronic supplementary material:**

The online version of this article (doi:10.1007/s00415-014-7241-y) contains supplementary material, which is available to authorized users.

## Introduction

Establishing what’s different about those who develop a disease as compared to those who remain unaffected (epidemiology) seems like it should be a fairly straightforward way of identifying clues to aetiology. In practice, however, such studies are surprisingly vulnerable to subtle biases that can easily generate false positive associations [4, 13]. Even when an exposure can be accurately measured and does not change over time, such as an individual’s genotype [or month of birth (MOB)], it is still possible for differences in ascertainment to result in apparently significant differences between cases and controls in the absence of any real effect, if the frequency of the exposure differs between sub-groups of the population considered [5, 31]. In genome-wide association screens, the large number of variants studied enables investigators to quantify and compensate for the influence of potentially confounding factors such as ancestry [17]. On the other hand, in studies considering individual risk factors (genetic or environmental), researchers cannot undertake such correction and instead are often forced to make simplifying assumptions, such as that within a given country exposure is likely to be uniform, and therefore that any confounding arising because of differences in how cases and controls are ascertained across the country is unlikely to be significant. For many risk factors this assumption is safe; for example, it certainly seems to be true for the vast majority of common genetic variants [3]. However, in the context of environmental risk factor analysis, the assumption of homogeneity of exposure has rarely been tested.

## Heterogeneity in the timing of birth

Although there are many local and personal factors that might influence an individual’s MOB, it seems reasonable to imagine that across the population in any given country these effects would likely average out; indeed, intuitively it feels unlikely that the probability of being born in any given month would vary between different parts of the same country, and equally unlikely that this probability might be significantly different in different years. In this context, it is unsurprising that the studies that have looked for association between multiple sclerosis and MOB have all assumed some degree of such homogeneity [2, 8, 11, 12, 16, 23–25, 27–29, 32, 33]. Unfortunately, it turns out that this assumption is invalid, and that confounding rather than biology has likely generated the apparent associations that have previously been reported [9]. The fact that MOB is extremely heterogeneous in the general population is well known in the anthropology literature [7, 15, 19–22], but seems to have gone largely un-noticed by those studying MOB as a potential risk factor in multiple sclerosis.

**Fig. 1:**
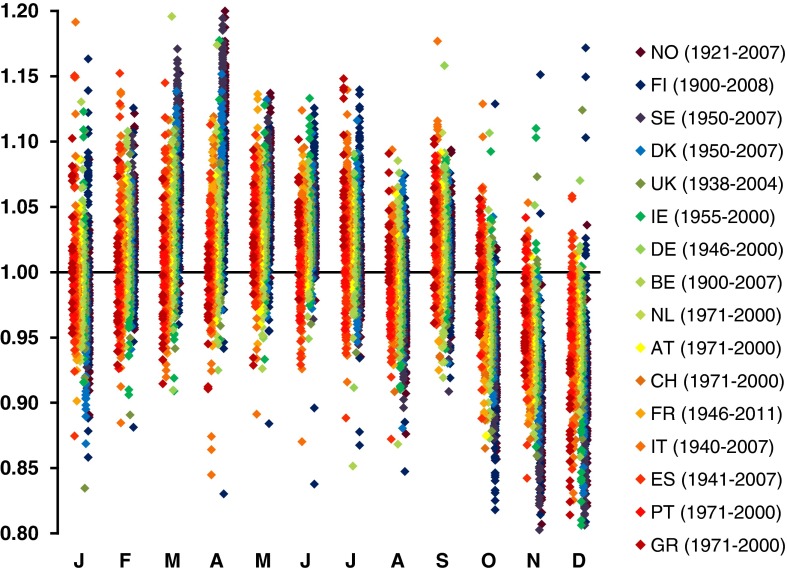
Year and country-specific month of birth records from 16 European countries (Greece, Portugal, Spain, Italy, France, Switzerland, Austria, Netherlands, Belgium, Germany, Ireland, UK, Denmark, Sweden, Finland and Norway) [9]. The *x* axis shows the months of the year (coded by their *first letter*), while the *y* axis shows the ratio between the observed number of births in a month and the number of births that would have been expected if birth rate had remained constant throughout the corresponding year. Ratios were calculated allowing for the length of each month and for leap years, but for simplicity are plotted assuming the length of each month is equal. For clarity, the country specific records are offset on the *x*-axis according to latitude and heat coded. The *legend* indicates the range of years included and the two letter country code. A total of 824 year and country-specific records are shown (including over 270 million births)

**Fig. 2:**
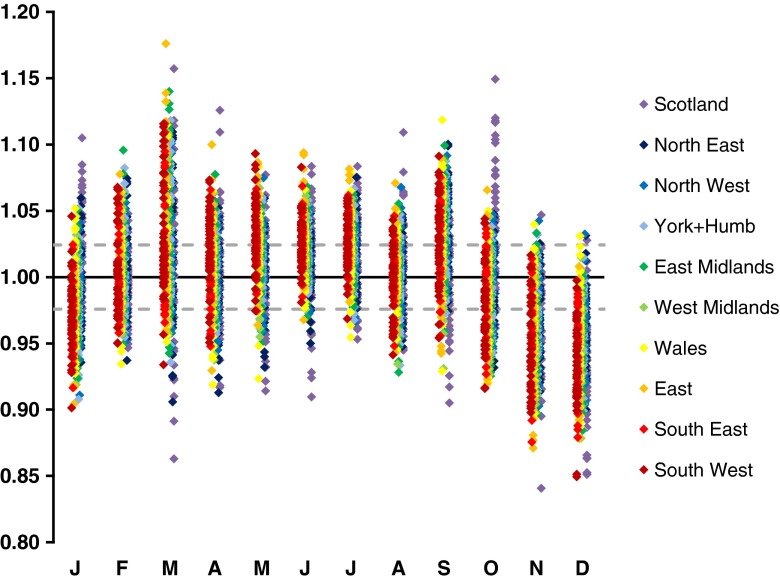
MOB records for individual UK Government Office Regions (GOR) over the period 1965–2008. For administrative purposes, the UK is currently divided into 11 regions (each with a roughly equivalent population, circa 6 million); however, in the past, London was included as part of the South East and only ten regions were considered [9]. Since some records predate this spilt, we have considered London and the South East together in all records, and only considered the ten GOR. Again for clarity, individual regions are heat coded and slightly offset on the *x*-axis. Nota bene until very recently Scottish statistics were based on the month of birth registration rather than the actual month of birth. A total of 440 year and GOR-specific records are shown (including over 32 million births, data obtained from UK National Statistics Office-www.statistics.gov.uk). The *dotted lines* indicate the 95 % confidence interval that would be expected for these regions (based on their population size) if the underlying birth rate were constant

If the underlying birth rate in a country remained constant over time, we would only expect random fluctuations in the ratio between the observed and the expected number of births seen in any given month; with the 95 % confidence interval on this ratio being 0.97–1.03 in a country like Norway (population circa 5 million) and 0.99–1.01 in a country like the UK (population circa 60 million). Surprisingly, this ratio shows much greater variation. Figure 1 shows the actual ratio of observed to expected births in each month present in 824 year and country-specific MOB records obtained from the national statistics available online from 16 European countries (as we described previously [9]). Each of these 824 records is statistically significantly different from that expected assuming a constant birth rate, with all but three records remaining significant even after stringent Bonferroni correction (i.e. having *p* < 6 × 10^−5^). Even when comparing each record with the number of expected births calculated by averaging across all the records for the corresponding country (ignoring 1/29 of the February births in leap years so that all records considered are based on 365 days per year), 807/824 records are significantly different (735/824 after Bonferroni correction). Furthermore, all 824 of the records include at least one spring month (March, April, May) where there is an excess in the birth rate and/or at least one winter month (November, December or January) showing a deficit. In more than 70 % (596/824) of the records, at least one of these differences is statistically significant. Some 88 % of records are significantly different from the preceding year in the same country. Figure 2 shows that even within a country there is marked and highly significant heterogeneity in birth rate, with 99 % of records (437/440) for individual UK Government Office Regions being significantly different from that expected if birth rate were constant and 85 % of records (374/440) are significantly different from the average across all records from the corresponding region. These data confirm that birth rate is subject to marked seasonal variation and allows us to unequivocally reject the assumption that birth rate is homogeneous.

## Heterogeneity in the distribution of multiple sclerosis

The frequency of multiple sclerosis also shows considerable variation both between [14, 26] and within countries [1, 30, 34]. Because of this variation, even comprehensive case collections that have been established through national registries in a single country will inevitably include a disproportionate number of individuals from some regions (those with higher prevalence) and an under-representation of those from other regions (those with lower prevalence). Furthermore, because the incidence of multiple sclerosis is age-dependent [6], any set of prevalent cases will necessarily be heterogeneous with respect to year of birth; including an excess of middle-aged individuals (the peak risk group) and smaller numbers of very young or much older individuals. The sort of variation in regional origin and year of birth that would be expected within a set of cases from a given country is illustrated in the Supplementary Figure S1. Cohorts collected through the efforts of interested researchers are likely to be even less representative of the country as a whole, as such collections are invariably biased in favour of prevalent cases from regions local to the interested investigator(s).

## Mismatching of cases and controls

**Fig. 3:**
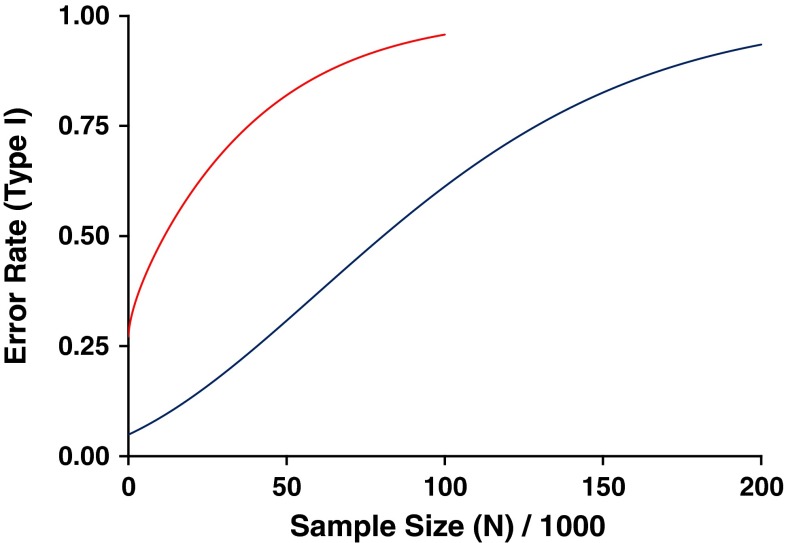
Conservative estimate for the type I (false positive) error rate expected in MOB studies of differing size (*N* = the number of cases = the number of controls, in thousands), reproduced with permission from our original publication [9]. The *lower curve* indicates the probability of identifying any month showing a significant difference; *p* value < 0.0042 (= 0.05 Bonferroni corrected for the number of months), while the *upper curve* indicates the probability of seeing a nominally significant excess in at least one spring month and/or a nominally significant deficit in at least one winter month

Given that seasonality of birth and multiple sclerosis are both highly heterogeneous with respect to geography and time, any mismatching for these extraneous variables between cases and controls has the potential to generate a spurious difference in the MOB pattern between these groups; this apparent association only reflecting differences in the regional and temporal origin of the two groups, rather than any genuinely causal effect. While we would expect such differences to get smaller as sample size increases, they will not tend toward zero unless cases and controls are fully matched for regional origin and year of birth. Since the significance of any given difference tends to increase as sample size increases, the likelihood of seeing a significant false positive association as a result of mismatching actually increases as sample size increases [18]. Thus, although calculating the number of births expected in each month by averaging over available national birth statistics is mathematically easy and seems intuitively reasonable, the resulting estimates are only appropriate if the case collection tested has the same regional and temporal distribution. Unfortunately, none of the studies that have assessed MOB as a risk factor for multiple sclerosis have adequately matched their cases and controls for both regional origin and year of birth, making it highly likely that the reported associations are false positives. Figure 3 shows a conservative estimate for the rate of false positive association expected, assuming that controls are based on averaged national statistics, while case recruitment is weighted by the typical prevalence and year of birth data shown in supplementary figure S1 and as shown previously [9].

## Why do the results from MOB studies in multiple sclerosis seem to be consistent?

**Fig. 4:**
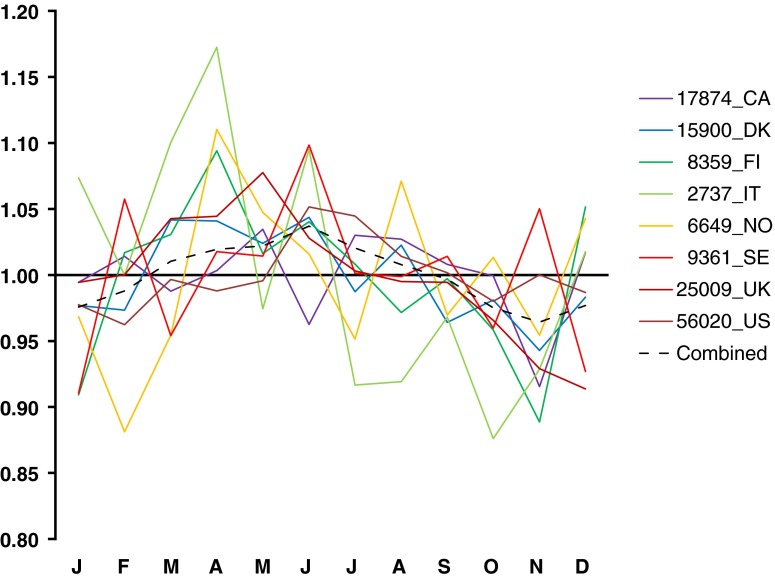
Country specific multiple sclerosis MOB data from previously published reports [2, 8, 9, 12, 16, 23, 25, 29, 32, 33]. The *x*-axis indicates the month of the year (as in Fig. 1), while the *y* axis indicates the ratio of observed to expected birth in each month as previously reported, (see individual publications for the details of which national statistics were used to calculate the expected numbers). The *legend* indicates the number of cases studied together with the standard two letter country code. The *dotted line* indicates the ratio based on combining all the available data; the tendency to excess in spring and deficit in winter is apparent, as is the extreme heterogeneity between the studies

Although it remains unclear exactly what factors drive the extensive variation in MOB that is apparent in national birth statistics, it is well established that there is a highly significant correlation between latitude and birth rate that is positive in spring months (March, April, May) and negative in winter months (November, December or January) [19, 21, 22]. Furthermore, there is significant evidence that these correlations have declined over time, such that there is much less seasonality in birth in today’s developed world than was apparent previously [7, 15, 20, 21]. It has been suggested that photoperiod (the hours of daylight in a day) might be responsible for the correlation with latitude, and it has also been suggested that perhaps the decline in these latitudinal gradients over time reflects our increasing ability to control our environment (through lighting and heating), and thus disconnect ourselves from the influence of such seasonal variables [10]. While these latitudinal and temporal correlations only account for a small fraction of the observed variation in birth rate, they do mean that there is an inevitable tendency for cases (which are generally older and more northern than the full set of individuals included in population-based birth statistics) to show higher rates of birth in spring months and lower rates of birth in winter months [9]. Coupled with the latitudinal gradient in the frequency of multiple sclerosis, these trends explain the rather superficial consistency in the MOB pattern considered typical of multiple sclerosis. These correlations favour the emergence of an apparent increase in risk during spring and reduction in risk during winter (see Fig. 4). Figure 3 shows a conservative estimate for the false positive replication of the MOB pattern considered typical of multiple sclerosis; i.e. a nominally significant excess in at least one spring month (March, April, May), and/or a nominally significant reduction in at least one winter month (November, December or January). These false positive rates are conservative, as they ignore the inevitable heterogeneity in MOB present within each of the individual Government Office Regions.

## What level of matching is required?

As the population in a country is divided into smaller and smaller regional groups, the variance in the ratio of observed to expected births per month that results from random sampling will increase. At some point, this sampling variance will overwhelm the systematic effects driving the variation evident at the whole country level, and no heterogeneity in MOB will be apparent within such groups. Based on the variance in the ratio of observed to expected births per month evident at the whole country level (Fig. 1), we would anticipate that there would be little or no power to demonstrate heterogeneity in MOB in populations where the average total birth rate is < 1,200 per year. Supplementary Figure S2 shows the MOB data from the 9-year period 2000–2008 for the 195 Local Authority regions from England and Wales that had an average annual birth rate of more than 1,200 over that time period. Since each of these local authorities has a population of only approximately 200,000, the range of ratio values is much greater than in the GOR (population circa 6 million, Fig. 2), while the power to demonstrate these differences as significant is limited. In fact, 15 % of these records show statistically significant evidence for seasonality. Given the decline in seasonality known to have occurred over the last century, it seems highly likely that local authority data from previous decades would be even more highly structured. These data confirm that MOB is heterogeneous down to the Local Authority level, and suggest that population statistics of corresponding resolution would likely be necessary to adequately control for year of birth and regional origin in an analysis of MOB as a risk factor in multiple sclerosis. It is unlikely that such detailed data exist in most countries. The extensive range of possible values for the ratio of births evident in the Supplementary Figure S2 also explains why studies considering cases collected in a single centre are likely to identify effects that are apparently larger than those seen in studies considering nationally recruited cases [2]. The greater variance in the smaller denominator population from which the cases are drawn necessarily exceeded anything seen as a result of systematic effects.

Using unaffected siblings as a source of controls is a logical way to try and reduce the confounding due to differences in regional origin [12, 27, 33]; however, these special individuals are again drawn from a much smaller denominator population, and of course are necessarily un-matched for year of birth. In multiple sclerosis, results using such controls have been inconsistent with each other and are too few in number to enable any confident assessment.

## Conclusion

Although intuitively it seems reasonable to conclude that comparing the MOB seen in a group of cases with that expected based on averaged national birth statistics from the same country should provide a robust way of assessing the role of MOB as a risk factor, in reality, the extensive and highly significant variation in MOB that is present in the general population means that the inevitable heterogeneity in cases with respect to regional origin or year of birth frequently generates false positive association [9]. Unfortunately, the high rate of false positive association likely to arise as result of this under-recognised structure means that it is very likely that previous reports of association are false positive, and that in fact there is no MOB association in multiple sclerosis. These observations underline how easily false positive associations can arise when a tested exposure is wrongly assumed to be homogeneous. Many environmental factors are highly heterogeneous within the general population (e.g. smoking and vitamin D levels), raising the possibility that hidden structure could also undermine the testing of these variables. These observations serve to remind us that controlling for confounding needs to be as comprehensive in the analysis of candidate environmental risk factors as it is in genetics; exactly how this could be done is not immediately clear.

## Electronic supplementary material

Below is the link to the electronic supplementary material.

Supplementary material 1 (DOC 335 kb)
