# Supplementary material for: Confounding in association studies: month of birth and multiple sclerosis
Source: J Neurol. 2014 Jan 12;261(10):1851–6. doi: 10.1007/s00415-014-7241-y (PMC4192561; doi:10.1007/s00415-014-7241-y)
Supplement: Supplementary file 1 — Supplementary material 1 (DOC 335 kb) [file 415_2014_7241_MOESM1_ESM.doc]

**Title:** Confounding in association studies: month of birth and multiple sclerosis

**Authors:** Barnaby Fiddes, MRCP,^1^ James Wason, PhD^2^, and Stephen Sawcer, FRCP PhD^1^

**Affiliations:**

^1^University of Cambridge, Department of Clinical Neurosciences, Box 165, Cambridge Biomedical Campus, Hills Road, Cambridge, CB2 0QQ, UK

^2^Medical Research Council Biostatistics Unit, Cambridge, CB2 0SR, UK

**Corresponding Author:** Stephen Sawcer

University of Cambridge, Department of Clinical Neuroscience, Addenbrooke’s Hospital, Hills Road, Cambridge, CB2 0QQ, UK

Phone: +44 1223 216073

FAX: +44 1223 336941

email: [sjs1016@cam.ac.uk](mailto:sjs1016@cam.ac.uk)


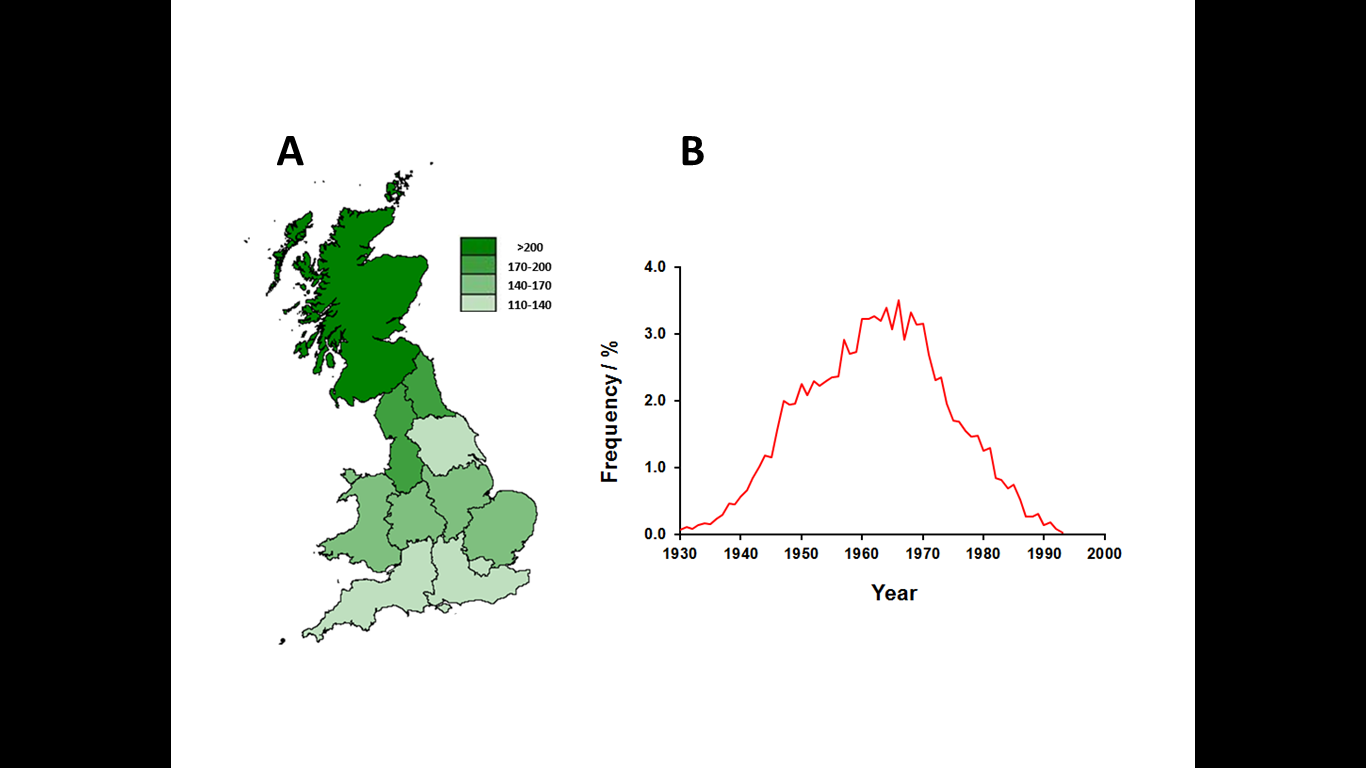


**Figure S1.** Heterogeneity in the distribution of multiple sclerosis. Panel A is a heat map illustrating the varying prevalence of multiple sclerosis in different UK GOR (the prevalence ranges from >200 per 100,000 in Scotland down to 120 per 100,000 in the south of England). Panel B shows the frequency of cases with respect to year of birth as seen in the 12198 cases from our data base that we have previously reported (Fiddes et al. 2013 Ann Neurol 73: 714-20).


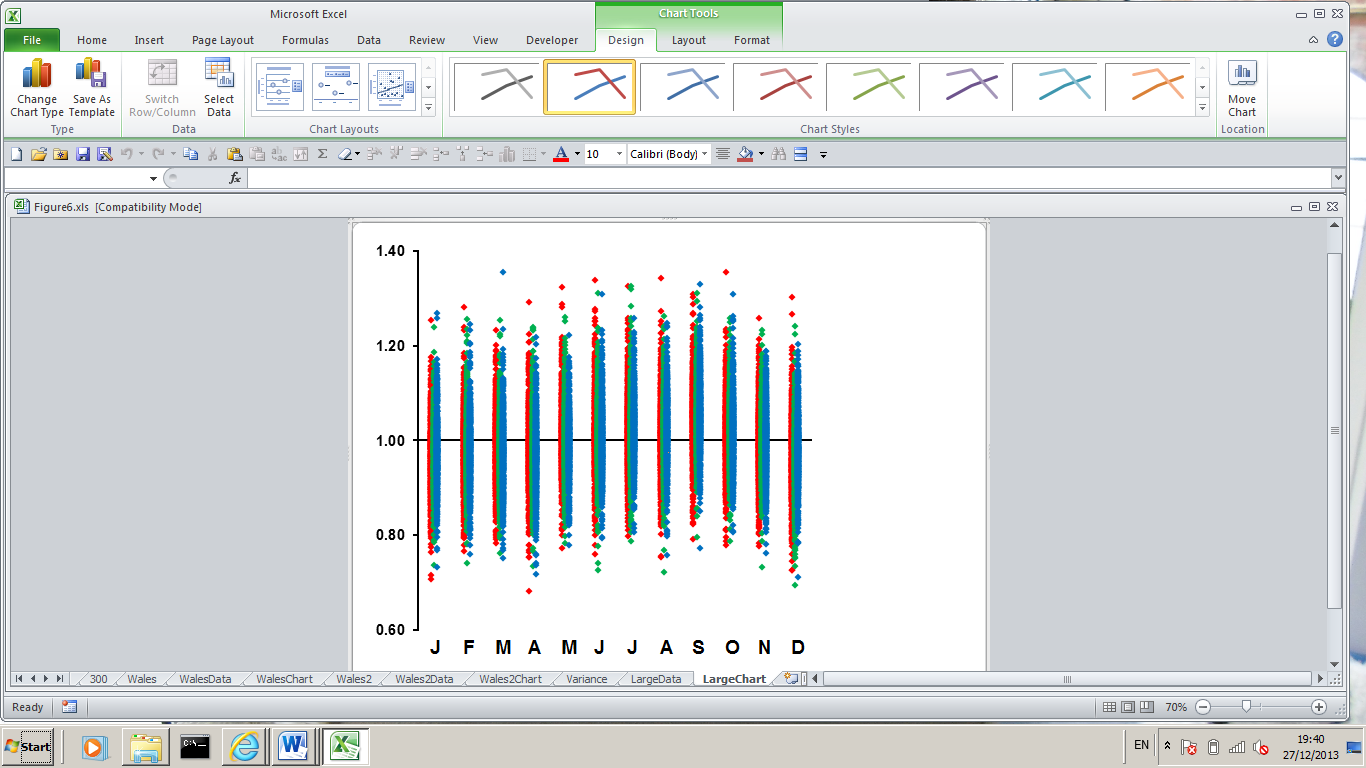


**Figure S2.** MOB data from 195 UK local authorities for the period 2000-2008 (a total of 1755 records obtained from UK National Statistics Office - www.statistics.gov.uk). As in figure 1 the x-axis indicates the month and the y-axis the ratio of the observed to expected births calculated assuming a constant birth rate. In this recent epoch there is very little seasonality. Note that the scale on the y-axis is double that in the other figures, in keeping with the greatly increased variance expected in smaller populations. Data from local authorities in the south (red), central part of the country (green) and north (blue) are plotted slightly offset to improve clarity.
